# How to mitigate the inhibitory effect of organizational inertia on corporate digital entrepreneurship?

**DOI:** 10.3389/fpsyg.2023.1130801

**Published:** 2023-03-09

**Authors:** Wei Li, Wei Chen, Qingdan Pang, Jianmin Song

**Affiliations:** ^1^School of Management, Chongqing University of Technology, Chongqing, China; ^2^Institute of Digital and Intelligent Management, Chongqing University of Technology, Chongqing, China; ^3^School of Economics and Business Administration, Chongqing University, Chongqing, China

**Keywords:** corporate digital entrepreneurship, organizational inertia, digital capability, entrepreneurial culture, institutional support, strategic alliance

## Abstract

As a novel way for incumbent firms to discover and utilize entrepreneurial opportunities in the digital era, corporate digital entrepreneurship (CDE) is significant for realizing digital transformation through dealing with organizational sclerosis and bureaucratization. Previous studies have identified the variables having positive effects on CDE and put forward practical solutions to promoting CDE. However, the majority of them have ignored the variables having negative effects on CDE and how to mitigate the inhibitory effects. In order to fill the research gap, this study investigates the causal relationship between organizational inertia (OI) and CDE and examines the moderating roles of internal factors such as digital capability (DC) and entrepreneurial culture (EC) as well as external factors such as institutional support (IS) and strategic alliance (SA). Based on multiple linear regression (symmetric) and fuzzy-set qualitative comparative analysis (asymmetric) using survey data from 349 Chinese firms, the results demonstrate that OI has a significant negative effect on CDE. In addition, DC, EC, and SA play negative moderating roles in the relationship between OI and CDE, which means that they could reduce the inhibitory effect derived from OI when incumbent firms implement CDE. Moreover, dividing OI into three dimensions discovers that the moderating roles of DC, EC, and SA present different features. This study enriches the literature on corporate entrepreneurship and provides valuable practical implications for incumbent firms to achieve successful CDE by revealing how to overcome the inertia deeply embedded in organizations.

## 1. Introduction

Over the past decades, a large body of literature has reached a consensus that corporate entrepreneurship could be highly beneficial to shaping sustainable competitive advantages for incumbent firms (Miller, 1983; Zahra, 1991; Chen et al., 2021). In the digital age, when digital technology has penetrated into such activities as production, marketing, communication with colleagues and cooperation with partners, corporate entrepreneurship is undergoing tremendous changes. Generally, incumbent firms are keen on applying emerging digital technologies such as artificial intelligence, blockchain, cloud computing, and big data to entrepreneurial activities, realizing digital technology-based corporate entrepreneurship, called corporate digital entrepreneurship (CDE) (Kraus et al., 2019; Chatterjee et al., 2021; Li, 2021; Sahut et al., 2021). A typical example of CDE is Haier Group, a well-known traditional manufacturing company in China, which has successfully realized digital transformation by adopting digital platforms to launch second entrepreneurship (Nambisan et al., 2019; Li et al., 2022).

Unfortunately, although CDE has been increasingly important for individual companies and macro-economic transformation, the academic community has not paid enough attention to it. First, the existing literature has not reached a broad consensus on the definition of CDE. As a result, many studies confused the conception of CDE with corporate entrepreneurship and digital entrepreneurship (Li, 2021; Li et al., 2022). Li (2021) particularly emphasized that CDE is markedly different from traditional corporate entrepreneurship and digital entrepreneurship in terms of subject, form, and structure. Corporate entrepreneurship usually focuses on the strategic adjustment and dynamic behaviors by incumbent firms for taking immediate response to environmental changes. Generally, corporate entrepreneurship does not include the utilization of digital technologies (Zahra, 1991; Heavey et al., 2009; Wang et al., 2015). Digital entrepreneurship stresses that entrepreneurial teams or individuals use digital technologies on digital platforms or in digital ecosystems to implement entrepreneurial activities without involving entrepreneurial behaviors at the organizational level (Geissinger et al., 2019; Soluk et al., 2021). As a relatively new concept and issue, CDE mainly stems from the concepts of corporate entrepreneurship and digital entrepreneurship and focuses on digital entrepreneurship at the organizational level rather than the individual level (Li, 2021; Li et al., 2022). In other words, CDE is the result of the evolution of corporate entrepreneurship in the ubiquitous digital era. Overall, the published literature has not developed a lucid definition and a reasonable theoretical framework for the concept of CDE.

Second, the overwhelming majority of previous studies usually have discussed the antecedent variables having positive effects on CDE, such as top management team (Behrens and Patzelt, 2016; Chen et al., 2021), knowledge acquisition (Bojica and Fuentes, 2012), institutional support (Soluk et al., 2021), but they have largely neglected to identify critical negative antecedent factors from the perspective of reducing the failure rate. Unlike new ventures, incumbent firms are often constrained by various factors when carrying out entrepreneurial activities, such as asset specificity, organizational rigidity, path dependence, and aversion to risk. These factors make it difficult to take immediate and flexible responses to unexpected environmental and technological changes as new ventures do (Jafari-Sadeghi et al., 2021; Moradi et al., 2021). Instead, incumbent firms have to overcome organizational inertia (OI) in product management, organizational structure, and human capital to conduct CDE (Svahn et al., 2017; Li, 2021). Thus, OI is considered to be an essential organizational factor restricting CDE. Nevertheless, previous studies still fail to test the negative impact of OI on CDE.

Third, since OI is an essential factor with a potential inhibitory effect on CDE, existing research still has failed to advance the knowledge of how to help incumbent firms reduce its negative influence. In fact, organization and environment alignment theory has pointed out that firms could optimize their management activities and organizational behaviors by considering boundary conditions (Sakhdari, 2016). The organization and environment alignment theory holds that with the rapid development of technology, organizational boundaries are rapidly penetrating and blurring. Therefore, in the business ecosystem, the competition pattern is no longer between individual enterprises, but between alliances. Only fast, flexible, innovative and environmentally sensitive organizations can obtain continuous competitive advantages at the organizational boundary. On the other hand, the complex and volatile super-competitive environment also poses severe challenges to all elements within the enterprise. As for incumbent firms, strategic alliance (SA), institutional support (IS), digital capability (DC), and entrepreneurial culture (EC) are usually seen as significant external and internal moderators. However, there is scarce research attaching importance to integrating these conditions into the holistic framework of CDE. Therefore, it is also valuable and meaningful work to discover and mitigate the inhibitory effect of OI on CDE, especially at the stage of ubiquitous digital transformation.

To fill these three research gaps, this study puts forward three research questions: RQ1: What is the notion of CDE? RQ2: To what extent does OI hinder CDE? RQ3: To what extent do DC, EC, IS, and SA moderate the negative relationship between OI and CDE? In order to answer these questions, this study develops a theoretical model based on the perspective of contingency theory. First, this study initially conceptualizes and operationalizes the notion of CDE and divides it into three dimensions: digital strategy generation, digital innovation, and digital business development. Second, as the dominant logic of past knowledge and experience paths, OI with three dimensions such as insight inertia, action inertia, and psychological inertia, is the independent variable in the theoretical model (Shimizu and Hitt, 2005; Huang et al., 2013; Ashok et al., 2021). Third, the external conditions include two variables: SA and IS. SA refers to the cooperative relationship between different enterprises (Cacciolatti et al., 2020; He et al., 2020); IS refers to the institutionalized resource and guarantee provided by national administrative agencies such as government and regulatory agencies for enterprises (Oliver, 1997; Smirnova, 2020; Soluk et al., 2021). Fourth, this study selects DC and EC as internal conditions. DC is defined as the capability to use digital technologies to s acquire, manage, understand, integrate, communicate, evaluate and create various forms of data safely and appropriately (Proksch et al., 2021); EC can be regarded as an essential category of organizational culture which is conducive to stimulating organizational vitality and individual creativity (Buccieri et al., 2020). Fifth, this study draws on 349 survey data from Chinese firms and uses multiple linear regression and the fsQCA methods to test our research hypotheses.

Threefold theoretical contributions are generated in this paper. First, this research plays a pioneering role in putting forward the notion of CDE and identifying its conceptual structure of CDE. Specifically, this study argues that CDE differs from corporate entrepreneurship and digital entrepreneurship in the subject, forms, and structure embedded in almost all parts of the value chain. Instead, CDE could be regarded as a creative combination of corporate entrepreneurship and digital entrepreneurship and divided into three dimensions: digital strategy generation, digital innovation, and digital business development. The exploration into the definition and conceptual structure of CDE enriches the managerial literature on corporate entrepreneurship in the digital era. Second, this study fills the gap that previous studies ignore in discussing inhibiting antecedents for CDE by linking OI with CDE. Compared with the studies that mainly discuss positive antecedent factors, this study discovers a new antecedent variable negatively associated with CDE. Therefore, this study not only enriches the literature on identifying important negative antecedent variables but also provides theoretical and empirical evidence for achieving digital transformation, which could reduce the possibility of unsuccessful entrepreneurship. Third, this study also answers the question, “How do incumbent firms take advantage of internal and external conditions to mitigate the inhibitory effect of OI on CDE.” By testing moderating effects of DC, EC, IS, and SA, this study extensively understands when the inhibition effect of OI on CDE would get weak. These findings provide theoretical guidance on fueling CDE by matching different types of OI with different boundary conditions, enriching the managerial literature on CDE.

The remainder of this article is organized as follows: Section 2 elaborates on the theoretical background. Section 3 puts forward our hypotheses. Section 4 introduces the samples, measurements, and statistical techniques. Section 5 reports the results of regression and fsQCA. Section 6 discusses the findings and puts forward theoretical contributions and implications. Furthermore, the limitations and future directions are also mentioned in section 6. The concluding section summarizes the main contributions of this study.

## 2. Theoretical background

### 2.1. Corporate digital entrepreneurship

The conception of CDE stems from corporate entrepreneurship and digital entrepreneurship. It can be defined as the value-creating process in which incumbent firms adopt digital technologies to discover and utilize new business opportunities for shaping, maintaining, or strengthening competitive advantages (Ghosh et al., 2021; Li, 2021 pp. 44). Since the notion of corporate entrepreneurship was proposed for the first time by Miller (1983), early research has largely focused on various entrepreneurial activities carried out by venture capital teams in respective organizations and considered it as the endogenous power that could promote the continuous development and expansion of firms (Antoncic and Hisrich, 2001; Hornsby et al., 2009; Wang et al., 2015). Therefore, corporate entrepreneurship primarily reflects the dynamic adjustments by incumbent firms for achieving the alignment of organization and environment (Zahra, 1991; Heavey et al., 2009; Li, 2021).

With the rapid development of digital technologies, traditional corporate entrepreneurship is confronted with dramatic changes in entrepreneurial philosophy, model, and approach (Nambisan et al., 2019; Arfi and Hikkerova, 2021; Dana et al., 2022a). One notable feature is that a large number of advanced digital technologies (e.g., artificial intelligence, blockchain, cloud computing, and big data) are widely and deeply integrated into modern entrepreneurial activities, which has a profound impact on digital entrepreneurship (Kraus et al., 2019; McAdam et al., 2019; Soluk et al., 2021). For example, digital technologies realize the remote communication between producers, service providers, traders, and end users, which greatly lowers the thresholds and remove the obstacles to entrepreneurship (Srinivasan and Venkatraman, 2017). Moreover, digital technologies could promote research and development collaboration within and across organizations, stimulating more value-creating activities across borders (Ross and Blumenstein, 2015). Furthermore, compared with the traditional distribution of products, digital technologies such as digital platforms and media could help companies to sell products with higher efficiency and low cost, which would greatly inspire those mature firms who have remarkable resource advantages to promote the digitalization of traditional business (Nambisan, 2017; Dana et al., 2022b). Obviously, the existing studies on digital entrepreneurship have paid more attention to entrepreneurial activities that are mainly conducted by individuals and teams on digital platforms or in digital ecosystems (Geissinger et al., 2019; Soltanifar et al., 2021; Soluk et al., 2021).

**Figure 1:**
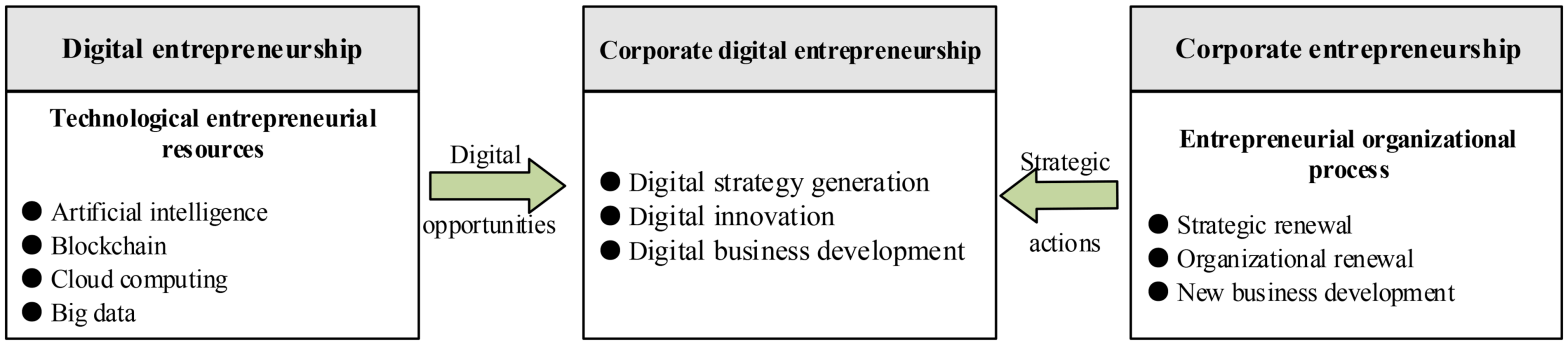
The framework of CDE. This figure is drawn by the authors based on Li (2021).

By contrast, CDE is a combination of corporate entrepreneurship and digital entrepreneurship and focuses on digital entrepreneurial activities at the organizational level (Li, 2021; Li et al., 2022). For example, incumbent firms introduce emerging technological products or services into their own business through CDE and then create brand new business models and development paths using their technical knowledge, business expertise, and ecological relationships with partners (Nambisan et al., 2019). In other words, CDE reflects the interaction between “grabbing digital opportunities driven by technological entrepreneurial resources” and “implementing strategic plans influenced by entrepreneurial organizational process” (Li, 2021). From digital transformation in traditional manufacturing firms such as Midea Group and Haier Group to digital innovation in emerging Internet giants such as Tencent and ByteDance and to digital business development in traditional service-oriented companies such as Ping-An Insurance^1^ and JINKE Service,^2^ these incumbent firms have fully incorporated digital technologies into their business for establishing and maintaining competitive advantages. Based on these arguments, this study follows the viewpoint of Li (2021) and divides CDE into three dimensions: digital strategy generation, digital innovation, and digital business development (see Figure 1). The digital strategy generation reflects the requirements of enterprises to carry out strategic renewal around digital strategy. Digital innovation focuses on all kinds of innovation activities enterprises carry out using digital technology within the organization. Digital business development emphasizes the development of new products or services based on digital technologies.

### 2.2. Organizational inertia

Previous studies have pointed out that organizations are not willing to change their systems and structures which have been stable and accepted by the majority of the members (Hannan and Freeman, 1984; Shimizu and Hitt, 2005). With the development and expansion of the organization, there would be an increasing tendency for the members to solidify and stereotype the management rules, business process, and even psychological model because they want to avoid risk and maintain the present situation. In other words, organizations always have a heavy inclination to cope with environmental changes along the old paths, which is called OI (Godkin and Allcorn, 2008; Mikalef et al., 2021). Therefore, OI is generally regarded as an endogenous organizational power preventing organizations from changing and developing in a changeable environment such as worldwide digital transformation. This power prefers stability to uncertainty and change in terms of management, product, manufacturing, marketing, culture, and economic policies (Ashok et al., 2021; Fu et al., 2021). Prior studies have confirmed that OI could be divided into multiple dimensions such as structural inertia, cognitive inertia, resource inertia, insight inertia, and action inertia and has a “double-edged sword” effect on the growth of enterprises (Shimizu and Hitt, 2005; Fu et al., 2021; Zhen et al., 2021).

According to the arguments of Shimizu and Hitt (2005) and Huang et al. (2013), this study discusses the negative effects of OI on CDE from three dimensions: insight inertia, action inertia, and psychological inertia. Insight inertia indicates that organizations could not immediately detect and identify the critical changes in the competitive environment. In other words, there is a long time lag between the occurrence of changes and the awareness of changes and possible results. Action inertia refers to organizations’ tendency to defend the status quo. Therefore, they are usually unwilling to respond promptly to the changes even though they have realized that the threats are approaching. Psychological inertia refers to members’ psychological resistance to the changes and mental anxiety over the threats when they have been conscious of external pressure. As a result, they will show apparent indifference or fierce opposition to internal and external changes.

## 3. Hypotheses development

**Figure 2:**
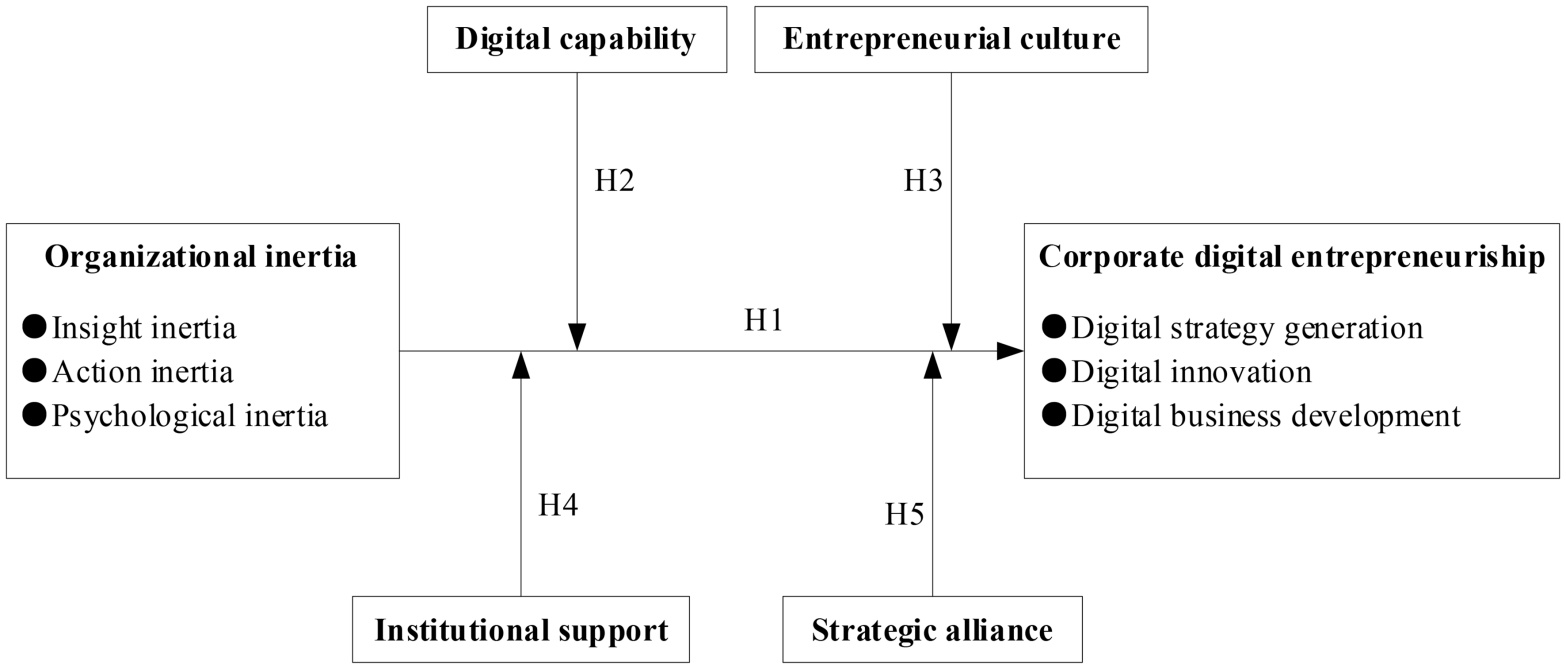
Theoretical model.

This study explores the negative effect of OI on CDE and discusses the moderating effects of external and internal conditions. Figure 2 displays the theoretical model.

### 3.1. The impact of OI on CDE

According to the viewpoint of Li (2021), CDE is a kind of entrepreneurial behavior that utilizes digital technologies and is full of change and innovation. However, traditional theories on strategic management argue that enterprises’ continuous development is always accompanied by increasingly solidified inertia (Ashok et al., 2021). OI is deeply ingrained in the interaction of the size, structure, process, and system of organizations and reflects the characteristics of their capabilities and behavioral patterns, which are difficult to change (Godkin and Allcorn, 2008; Fu et al., 2021). Therefore, OI is likely to prevent organizations from adjusting strategic orientation, changing business models, or promoting digital transformation (Hosseini et al., 2022). For instance, Huang et al. (2013) have found in their research on small and medium-sized enterprises that OI has a significant negative impact on open innovation and business model innovation.

More specifically, OI would inhibit incumbent firms from grabbing digital opportunities and promoting digital innovation because of short industrial insight, sluggish strategic actions, and ingrained psychological cognition. First of all, insight inertia is harmful to the perception of the recent trend in digital technologies. In other words, incumbent firms with serious insight inertia cannot quickly discover and identify the fleeting digital opportunities. Secondly, action inertia is detrimental to the immediate response to changes. Because the development of digital technologies and the competitive environment is fast and even unpredictable, action inertia would restrain incumbent firms from taking timely strategic measures to deal with the dilemma (Moradi et al., 2021). Finally, psychological inertia damages employees’ attitudes and behavior towards drastic changes in the external environment, such as the coming of digital trends, and internal conditions, such as the organizational system, strategy, and process. This kind of inertia would undermine their motivation to accept changes and ultimately have a negative effect on digital entrepreneurial activities in firms.

Overall, the solidified insight, behavior, and psychology would not only persuade incumbent firms to be satisfied with their current condition and to refuse organizational changes in the era of the digital economy but also make enterprises confronted with a wide range of rejection and resistance from employees when discovering and utilizing digital opportunities. Therefore, OI would bring many obstacles to CDE. According to the arguments above, we hypothesize that:

*H1*: OI is negatively associated with CDE.

### 3.2. The moderating effect of digital capability

Digital capability (DC) is defined as the ability to use digital technologies to create value (DeLone et al., 2018; Warner and Wäger, 2019). For firms, DC is a fundamental ability to improve customer experience, operational processes and business models (Westerman et al., 2012). At present, mainstream research has discussed DC from organizational and individual perspectives (Salamzadeh et al., 2021). Organizational digital capability refers to an enterprise’s skill, talent and professional knowledge in learning, absorbing, and applying a variety of digital technologies, which is usually reflected in the application of big data and digital platforms in product development and customer interaction (Proksch et al., 2021). Individual digital capability refers to an individual’s comprehensive ability to identify and use digital technologies in working, learning, and social participation, usually derived from an individual’s digital experience and technical expertise (Tzafilkou et al., 2022). For example, a published study discussed the two levels of digital capability: organizational IT capability and employee digital capability (Proksch et al., 2021). Overall, this study argues that digital capability, whether organizational or individual level, would have a profound impact on the application of digital technologies in an enterprise.

Specifically, DC could help incumbent firms to reduce OI by encouraging new working methods and strengthening organizational confidence by introducing new digital technologies. On the one hand, the digital organizational capability could enable incumbent firms to explore more entrepreneurial opportunities from cutting-edge digital technologies. After discovering new entrepreneurial opportunities in the digital area, incumbent firms could quickly identify and utilize digital technologies based on their digital capability to carry out innovation or creative activities (Warner and Wäger, 2019). Levallet and Chan (2018) proved that DC could promote firms’ improvisational behaviors and encourage them to use digital technologies to create value. On the other hand, employee digital capability could reduce personal anxiety about emerging digital technologies. In other words, employee digital capability could alleviate the attenuation of personal efficacy caused by new digital technologies and enhance the employees’ intrinsic motivation to accept and embrace digital applications and innovation. According to the arguments above, we hypothesize that:

*H2*: DC moderates the relationship between OI and CDE such at the relationship is less negative among with higher levels of DC.

### 3.3. The moderating effect of entrepreneurial culture

As an essential branch of corporate culture, entrepreneurial culture (EC) is highly related to psychological consciousness, consciousness values, and organizational atmosphere in entrepreneurial activities. From the organizational perspective, entrepreneurial culture is an enterprise’s holistic values and behaviors towards entrepreneurship. For example, members respond to environmental changes in innovative ways. This culture profoundly affects members’ initiative and creativity during identifying and utilizing entrepreneurial opportunities (Danish et al., 2019; Buccieri et al., 2020). There is no doubt that EC could be regarded as an essential factor in stimulating organizational vitality and enhancing individual innovative behavior, especially when enterprises are experiencing CDE (Fritsch and Wyrwich, 2017).

In addition, from the knowledge management perspective, EC could help organizations and employees proactively overcome OI by learning, practicing, and absorbing new knowledge about digital technologies and skills, promoting the spread and application of emerging digital technologies within organizations. For example, Ashok et al. (2021) found that an organizational culture that encourages experimentation and innovation could significantly reduce the negative influence of OI on the absorption of new knowledge. From the perspective of employee management, EC could inspire employees to engage in creative undertakings for meeting the development requirement of an organization (Danish et al., 2019). Furthermore, a healthy entrepreneurial culture could enhance employees’ innovative awareness and job engagement but also stimulate employees’ entrepreneurial cognition and intention. Therefore, entrepreneurial culture could mitigate and even eliminate the negative impact of the organization through two channels: expanding knowledge spread and inspiring individual initiative. According to the arguments above, we hypothesize that:

*H3*: EC moderates the relationship between OI and CDE such at the relationship is less negative among with higher levels of EC.

### 3.4. The moderating effect of institutional support

Institutional support (IS) refers to those laws, regulations, and policies formulated and issued by the central and local governments to guide and support the development of enterprises. It could not only reduce the negative impact caused by the flawed system in economic transition but also improve the allocation of social and economic resources and guide the industries and enterprises into correct development direction and operation behavior (Oliver, 1997; Hemmert et al., 2016; Ahsan et al., 2021). Considering the Chinese economy is experiencing the transition from a resource-intensive model to a technology-intensive model, the government plays an indispensable role in formulating and implementing proper industrial policies (He et al., 2019). In order to adjust and guide the development of industries and promote the growth of enterprises, the government will provide a wide range of institutional support for them (Zhang et al., 2017). For example, the government has released a series of regulations and policies to encourage enterprises to participate in digital transformation and digital entrepreneurship to develop digital economies. In other words, the external institutions could mitigate the negative impact of internal inertia.

From the perspective of a resource-based view, IS is an important environmental factor and a crucial strategic resource for enterprises (Oliver, 1997). Generally, the government provides a variety of IS, including tax exemptions, financial subsidies, technological platforms, and special business licenses. These institutional measures could consolidate enterprises’ resource endowment in applying digital technologies and reduce enterprises’ risk perception of innovation and change, which would be beneficial for suppressing the negative effects of action inertia and psychological inertia (Smirnova, 2020). Additionally, IS for incumbent firms means that government will provide more financial and technological resources and policy information about industrial trends, which could reduce the risk and pressure of CDE and enhance the creative awareness and behavior of employees. Therefore, IS could weaken the negative effects of insight inertia and psychological inertia. Ahsan et al. (2021) found that the perceived IS could significantly strengthen employees’ entrepreneurial persistence and enhances their self-efficacy and confidence to achieve successful CDE. Based on these arguments, we propose that:

*H4*: IS moderates the relationship between OI and CDE such at the relationship is less negative among with higher levels of IS.

### 3.5. The moderating effect of strategic alliance

A strategic alliance (SA) denotes a voluntary relationship between two and more independent enterprises to achieve their individual and mutual strategic goals (He et al., 2020). Previous studies have confirmed that SA plays a vital role in integrating external resources, reducing operating costs, and sharing the risk of innovation (Moghaddam et al., 2016; Bustinza et al., 2019). Formation, governance, utilization and performance are generally seen as four main topics in SA research (Dickson et al., 2006; Blevins and Ragozzino, 2018; Child et al., 2019). Considering that CDE emphasizes the acquisition of digital assets or business and is inspired by previous literature, this study discusses the moderating role of SA in mitigating the negative impact of OI on CDE from the perspectives of equity and non-equity (Cacciolatti et al., 2020).

The alliance and cooperation mechanism is a dispensable way to overcome OI during strategic changes (Cacciolatti et al., 2020). Compared with the non-equity alliance having the characteristics of relatively flexible and loose governance, an equity alliance is a more formal pattern constructed by joint venture or mutual shareholding between enterprises and partners. An equity alliance requires members to invest resources into the union and enforce investment proportion, resource allocation, and decision-making provisions. During CDE, an equity alliance with stricter governance is more conducive to helping incumbent firms overcome OI. On the one hand, a closer equity alliance could help enterprises acquire new knowledge and information on digital trends from external partners, reducing the negative impact of insight inertia.

Moreover, SA could propel the participants into positive responses and immediate actions because they should take the responsibilities and commitments in accordance with the agreement, which would relieve psychological inertia and action inertia (Joshi et al., 2019). On the other hand, SA between incumbent firms that focus on digital resources and digital business has a significant leverage effect. Enterprises could not only integrate and leverage more resources through limited resource input but also share the risk and cost of digital innovation and value creation. As He et al. (2020) suggested, the development of block chain, the Internet of Things, big data, and other emerging digital technologies has brought new ways to form strategic alliances, providing external opportunities like digital resources and digital technologies for incumbent firms and reducing their internal pressure of digital change and transformation. According to the arguments above, we hypothesize that:

*H5*: SA moderates the relationship between OI and CDE such at the relationship is less negative among with higher levels of SA.

## 4. Method

### 4.1. Samples and data collection

Like many studies involving digitization (Proksch et al., 2021; Lin et al., 2022), this study adopted a self-administrated survey method to collect cross-sectional data in China. As was mentioned by Straub et al. (2004) and Mikalef et al. (2020), this kind of survey method has many advantages in exploratory research and prediction theory testing. Considering that all the primary constructs in this study were at the organizational and strategic level, we only invited top managers who were familiar with the strategy and digital operations in the enterprise, such as the CEO, top managers, and senior managers (Yuan et al., 2021). Furthermore, in order to control the sample selection bias, we choose samples from four primary regions of China, such as Beijing and Tianjin (the north), Zhejiang and Shanghai (the east), Chengdu and Chongqing (the west), Guangzhou and Shenzhen (the south). Therefore, our samples could better represent the comprehensive development of Chinese firms. Finally, in order to collect highly valid data, we decided to cooperate with a professional company that has rich experience in conducting a market survey and questionnaire interview, which has become a very popular way to collect business and organization data in emerging economies (Yang et al., 2012; Wamba et al., 2020).

Before the formal distribution of questionnaires, a pretest was carried out to improve the questionnaire quality by one professor, one Ph.D. student, and two master’s degree students majoring in business and management. Specifically, we invited 100 senior managers who had experienced digital transformation or other digital practices in the past 3 years to complete the original version. Then, we distributed 100 questionnaires and received 98 valid questionnaires. Based on the feedback from 98 respondents, we deleted items whose corrected item-total correlations (CITC) were lower than 0.4, or the items whose square multiple correlations (SMC) were lower than 0.5, including four items in OI and six items in DC. Finally, we made a couple of modifications to the structure and language of the original design.

In the formal stage of the questionnaire survey, this research collected data in accordance with the following standard procedures. First, after adequate negotiation and communication with the candidates, we decided on the list of 800firms which were selected in the four regions in the same number of 200 and showed a strong willingness to participate in the survey. Second, paper or electronic questionnaires were distributed and returned immediately after finishing the questionnaires. Third, we carefully checked the questionnaires one by one and deleted the invalid questionnaires according to two fundamental criteria: (1) whether all the items were completed; (2) whether there were more than five same scores that were continuous. Overall, we delivered 800 emails and received 437 questionnaires in the survey period of more than 3 months. Finally, we got 349 valid questionnaires with a valid response rate of 43.63%. In addition, in order to alleviate common method bias, we collected data from firms in different regions and conducted ANOVA. Meanwhile, we also tested the nonresponse bias between early response and late response biases in this investigation. The results both showed that there were no significant differences between groups of cities and different groups of response. Table 1 reported the descriptive statistics of the samples.

**Table 1:** Descriptive statistics of sample.

Indexes	Category	Frequency	Per (%)	Indexes	Category	Frequency	Per (%)
Firm age (year)	<3.5 (not include)	38	10.89%	Firm size (Number of employees)	<50 (not include)	45	12.89%
	3.5–5	58	16.62%		50–149	92	26.37%
	6–10	83	23.78%		150–299	83	23.78%
	11–20	103	29.51%		300–499	88	25.21%
	>20	67	19.20%		>500	41	11.75%
Business scope	Product (only)	125	35.82%	Location	Chengdu and Chongqing	102	29.23%
	Product first/service second	63	18.05%		Beijing and Tianjin	97	27.80%
	Service first/product second	80	22.92%		Zhejiang and Shanghai	67	19.19%
	Service (only)	81	23.21%		Guangzhou and Shenzhen	83	23.78%

### 4.2. Measures

All measurements of each construct were extracted from previously validated literature. Unless otherwise indicated, all items were scored on a 5-point Likert-type scale ranging from 1 (strongly disagree) to 5 (strongly agree). Considering that the investigation was conducted in China, we followed Brislin (1980) translation-back-translation approach to prepare in English for translation into Chinese. All items of the scale can be found in Appendix I.

Organizational inertia (OI). OI was measured by thirteen items adapted from the scale developed by Huang et al. (2013). Among them, insight inertia contained four items; action inertia contained five items; psychological inertia contained four items. One sample item was “Our company rarely observes changes in the external environment.” We first conducted a pretest on this latent variable and eliminated four items with poor indicators, including one item in insight inertia, two in action inertia, and one in psychological inertia. The Cronbach’s alpha and AVE for the scale were 0.881 and 0.523, respectively (see Table 2).

**Table 2:** Results of reliability and validity.

Variables	Item	CITC	SFL	α	AVE	Variables	Item	CITC	SFL	α	AVE
CDE	DSG01	0.682	0.735	0.895	0.519	EC	EC01	0.626	0.748	0.827	0.533
	DSG02	0.636	0.723				EC02	0.613	0.732		
	DSG03	0.614	0.717				EC03	0.609	0.727		
	DSG04	0.607	0.708				EC04	0.582	0.713		
	DI01	0.692	0.738			IS	IS01	0.694	0.756	0.831	0.535
	DI02	0.683	0.723				IS02	0.672	0.729		
	DI03	0.654	0.715				IS03	0.621	0.723		
	DI04	0.638	0.708				IS04	0.615	0.718		
	DBD01	0.675	0.731			SA	SA01	0.675	0.736	0.833	0.525
	DBD02	0.636	0.726				SA02	0.649	0.725		
	DBD03	0.621	0.715				SA03	0.642	0.721		
	DBD04	0.607	0.705				SA04	0.636	0.716		
OI	II02	0.679	0.735	0.881	0.523	DC	DC03	0.683	0.748	0.869	0.519
	II03	0.654	0.724				DC05	0.672	0.724		
	II04	0.617	0.707				DC06	0.638	0.722		
	AI01	0.667	0.724				DC08	0.627	0.715		
	AI04	0.653	0.721								
	AI05	0.618	0.713				DC09	0.612	0.709		
	PI01	0.665	0.738								
	PI02	0.643	0.721				DC10	0.606	0.704		
	PI04	0.635	0.715								

CITC, corrected item-total correlation; SFL, standard factor loading; α, Cronbach’s alpha; AVE, average of variance extracted; CDE, corporate digital entrepreneurship; OI, organizational inertia; DC, digital capability; EC, entrepreneurial culture; IS, institutional support; SA, strategic alliance; we excluded II01, AI02, AI03, PI03, DC01, DC02, DC04, DC07, DC11, and DC12.

Corporate digital entrepreneurship (CDE). CDE was assessed using twelve items utilized in the pioneering research by Li et al. (2022). Each dimension (i.e., digital strategy generation, digital innovation, and digital business development) was measured with four items. Similarly, we conducted a pretest on this latent variable, suggesting that all items were ideal. One sample item was “Our company uses the digital technology to identify new target markets.” The Cronbach’s alpha and AVE for the scale were 0.895 and 0.519, respectively (see Table 2).

Entrepreneurial culture (EC). The measure of EC included four items adapted from the research by Buccieri et al. (2020). One sample item was “The company’s management team likes risky projects with the chance of a high return.” The Cronbach’s alpha and AVE for the scale were 0.827 and 0.533, respectively (see Table 2).

Digital capability (DC). Following the research by Proksch et al. (2021), we used twelve items to measure DC in terms of organizational IT and employees’ digital capabilities. One sample item was “Our company uses the most current IT infrastructure.” We also conducted a pretest on this latent variable to delete those items with poor indicators, including six items. The Cronbach’s alpha and AVE for the scale were 0.869 and 0.519, respectively (see Table 2).

Institutional support (IS). We measured IS using four items based on the original literature by Smirnova (2020). On sample item was “Implemented policies and programs that have been beneficial to the application and innovation of digital technologies in enterprises.” The Cronbach’s alpha and AVE for the scale were 0.831 and 0.535, respectively (see Table 2).

Strategic alliance (SA). We developed a four-item scale based on the idea of measuring the level of equity alliance by Cacciolatti et al. (2020) This scale focused on the degree to which enterprises conducted alliance governance through equity rather than contract. One sample item was “Our company acquires digital technology resources through investment or shareholding.” The Cronbach’s alpha and AVE for the scale were 0.833 and 0.525, respectively (see Table 2).

Control variables. Following the literature (Guo et al., 2016; Yuan et al., 2021), this study selected firm age, firm size, and business scope as three types of control variables. First, firm age was calculated by based on the number of years the enterprise has been established, including 1 = “<3.5 years,” 2 = “3.5–5 years,” 3 = “6–10 years,” 4 = “11–20 years,” and 5 = “>20 years.” Second, firm size was measured by the number of employees, including 1 = “<50,” 2 = “50–149,” 3 = “150–299,” 4 = “300–499,” and 5 = “>500.” Third, the business scope was measured by the characteristics of the products that firms provided, including 1 = “product (only),” 2 = “product first/service second,” 3 = “service first/product second,” and 4 = “service (only).”

### 4.3. Statistical techniques

We used both qualitative and quantitative methods to carry out this research. On the one hand, multiple linear regression emphasizes the net impact of the independent variable on the dependent variable, which helps us understand specific paths and directions between variables based on the rules of linearity, unifinality and additive effects (Woodside, 2013; Ciampi et al., 2021). On the other hand, fsQCA focuses on the asymmetric relationship between variables and the configuration effects based on complexity theory. This approach states that variables do not usually follow a strict causal relationship with each other. Instead, they are often multiple and concurrent (Rihoux and Ragin, 2009). Therefore, fsQCA explores the equifinality (different routes can generate the same outcome) and multifinality (identical elements can generate different outputs) between variables (Woodside, 2013; Ciampi et al., 2021). Since this research explores how to configure organizational factors to promote CDE, it is suitable to employ the fsQCA method.

## 5. Results

### 5.1. Reliability and validity

Reliability was assessed using Cronbach alpha and corrected item-total correlation (CITC). The results showed (see Table 2) that the value of Cronbach’s alpha of each construct ranged from 0.827–0.895, which exceeded the threshold of 0.7 (Fornell and Larcker, 1981; Ali, 2021). Meanwhile, the value of CITC of each item was greater than 0.5. These results demonstrated that the reliability of the scale was acceptable.

Additionally, this study evaluated the validity of the scale from two aspects: convergent validity and discriminant validity. To evaluate the convergent validity, we first conducted confirmatory factor analysis (CFA) for each variable. The results revealed that the model fits index of each variable reached the standard value: *χ*^2^/df was in the range of 1.0 to 2.0, RMSEA was lower than 0.8, CFI and AGFI were higher than 0.9, PGFI and PNFI were higher than 0.5. Meanwhile, each value of standard factor loading (SFL) was greater than the recommended value of 0.7 (Hair et al., 2011). Moreover, the AVEs of all variables exceeded the standard value of 0.5 (Fornell and Larcker, 1981), indicating that the scale had ideal convergence validity. To assess the discriminant validity, we used the Fornell-Larcker criterion. The result suggested (see Table 3) that the square roots of all AVEs of each construct were greater than the correlation coefficients of the row and column in which they were located (Fornell and Larcker, 1981), indicating that the measurement has good discriminate validity.

**Table 3:** The results of discriminate validity.

Variables	Mean	1	2	3	4	5	6	7	8	9
1. CDE	3.518	**0.721**								
2. OI	3.275	−0.301^***^	**0.723**							
3. DC	3.912	0.238^**^	−0.217^**^	**0.721**						
4. EC	3.836	0.192^*^	−0.158	0.108	**0.730**					
5. IS	4.125	0.098	−0.091	0.063	0.094	**0.731**				
6. SA	3.374	0.103	−0.078	0.125	0.089	0.047	**0.725**			
7. Firm age	2.543	0.117	0.137	0.086	0.169	0.103	0.163	—		
8. Firm size	2.746	0.163	0.195^*^	0.072	−0.105	0.018	0.135	0.172^*^	—	
9. Business scope	1.958	0.077	0.129	0.047	0.008	0.012	0.132	0.018	0.151	—

*p < 0.05, **p < 0.01, ***p < 0.001. Diagonal elements are the square roots of the AVE; The elements that appeared in the lower left are the Pearson Correlation Coefficient between constructs. CDE, corporate digital entrepreneurship; OI, organizational inertia; DC, digital capability; EC, entrepreneurial culture; IS, institutional support; SA, strategic alliance.

### 5.2. Hypothesis testing

We used multiple linear regression to test our hypotheses. Before conducting the regression, we assessed the multicollinearity between variables using the variance inflation factor (VIF) (García et al., 2015). The results showed that all VI*F* values were less than 2 (see Table 4), demonstrating that no obvious multicollinearity. Therefore, regression analysis could be further used to test hypotheses.

**Table 4:** The results of regression analysis of main effects.

Variables	CDE (1.537)					
	Model 1	Model 2	Model 3	Model 4	Model 5	Model 6
Firm age	0.118	0.112	0.109	0.102	0.099	0.092
Firm size	0.126	0.118	0.112	0.107	0.102	0.098
Business scope	0.097	0.089	0.085	0.083	0.077	0.072
OI (1.379)		−0.326^***^	−0.322^***^	−0.319^***^	−0.308^***^	−0.305^***^
DC (1.524)			0.205^*^	−0.202^*^	−0.192^*^	−0.188^*^
EC (1.482)				0.185^*^	−0.179^*^	−0.175^*^
IS (1.376)					0.106	0.102
SA (1.593)						0.186^*^
OI × DC			−0.237^**^	−0.231^**^	−0.225^**^	−0.217^**^
OI × EC				−0.253^**^	−0.248^**^	−0.239^**^
OI × IS					−0.137	−0.115
OI × SA						−0.294^***^
*R* ^2^	0.027	0.248	0.268	0.273	0.278	0.295
Adj-*R*^2^	0.022	0.235	0.247	0.261	0.268	0.272
Δ*R*^2^		0.221	0.241	0.246	0.251	0.268
*F* value	1.959	16.375^***^	17.095^***^	17.986^***^	18.153^***^	18.522^***^

*p < 0.05, **p < 0.01, ***p < 0.001. VIF values are in parentheses; CDE, corporate digital entrepreneurship; OI, organizational inertia; DC, digital capability; EC, entrepreneurial culture; IS, institutional support; SA, strategic alliance.

First, three types of control variables (i.e., firm age, firm size, and business scope) were included in model 1, suggesting that the impact of control variables on CDE was not supported. Second, we regressed OI with CDE in model 2 and the result showed that OI was negatively associated with CDE (*β* = −0.326, *p* < 0.001), demonstrating that H1 was supported. Third, model 3, model 4, model 5, and model 6 aimed to explore the moderating effects of DC, EC, IS, and SA. The results reported (see Table 4) that DC (*β* = −0.237, *p* < 0.01), EC (*β* = −0.253, *p* < 0.01), and SA (*β* = −0.294, *p* < 0.001) negatively moderated the relationship between OI and CDE. However, the interaction between OI and IS was not significant (*β* = −0.137, *p* > 0.05). These findings revealed that H2, H3, and H5 were supported, but H4 was not supported.

Since the negative effect of OI on CDE was confirmed, we further explored the moderating effects of three boundary conditions (i.e., DC, EC, and SA) on the relationship between three dimensions of OI and CDE. The results were reported in Table 5. First, the negative moderating effect of DC on the relationship between OI and CDE was mainly reflected in the level of action inertia (*β* = −0.223, *p* < 0.001). Second, the negative moderating effect of EC in this relationship mainly reflected in the level of action inertia (*β* = −0.297, *p* < 0.001) and psychological inertia (*β* = −0.261, *p* < 0.001). Finally, SA negatively moderated the relationship between insight (*β* = −0.318, *p* < 0.001) and action inertia (*β* = −0.245, *p* < 0.01) and OI.

**Table 5:** The results of regression analysis on interaction effect of moderators and OI.

Variables	CDE				
	Model 1	Model 7	Model 8	Model 9	Model 10
Firm age	0.118	0.113	0.107	0.097	0.085
Firm size	0.126	0.117	0.109	0.101	0.092
Business scope	0.097	0.085	0.081	0.075	0.071
Insight inertia (1.524)		−0.295^**^	−0.294^***^	−0.289^***^	−0.281^***^
Action inertia (1.482)		−0.338^***^	−0.327^***^	−0.315^***^	−0.311^***^
Psychological inertia (1.482)		−0.257^**^	−0.243^**^	−0.241^**^	−0.236^**^
DC			0.202^*^	0.193^*^	−0.189^*^
EC				0.183^*^	0.181^*^
SA					0.189^*^
Insight inertia × DC			−0.156	−0.153	−0.149
Action inertia × DC			−0.223^**^	−0.221^**^	−0.216^**^
Psychological inertia × DC			−0.147	−0.136	−0.127
Insight inertia × EC				−0.143	−0.139
Action inertia × EC				−0.297^***^	−0.293^***^
Psychological inertia × EC				−0.261^**^	−0.254^**^
Insight inertia × SA					−0.318^***^
Action inertia × SA					−0.245^**^
Psychological inertia × SA					−0.127
*R* ^2^	0.027	0.249	0.269	0.283	0.299
Adj-*R*^2^	0.022	0.236	0.248	0.261	0.278
Δ*R*^2^		0.222	0.242	0.256	0.272
*F* value	1.959	16.483^***^	17.139^***^	17.986^***^	18.953^***^

*p < 0.05, **p < 0.01, ***p < 0.001. VIF values are in parentheses; CDE, corporate digital entrepreneurship; DC, digital capability; EC, entrepreneurial culture; IS, institutional support; SA, strategic alliance.

### 5.3. FsQCA results

Although the regression analysis could help to understand the specific causal effect between different variables, a certain result is usually produced by a combination of various antecedents (Ragin, 2008). The method of fsQCA enabled to find out all the combinations of causal conditions that have the potential to result in a certain result (outcome) (Ciampi et al., 2021). In this research, the high level of CDE represented the outcome, while the causal conditions were the combinations of OI, DC, EC, IS, and SA. According to the procedure and principle suggested by Fiss (2011), we calibrated the variables by setting three anchor points, 75% represented full set membership, 50% represented the crossover point and 25% denoted no set membership. In addition, the calibration rules of CDE for non-high levels were opposite to the original set (see Table 6).

**Table 6:** Calibration anchors for each variable.

Variables		Target set	Anchors		
			Full membership	Crossover point	Full non-membership
Condition variables	OI	High OI	4.824	3.756	2.93
	DC	High DC	4.531	3.972	2.823
	EC	High EC	4.328	3.077	2.193
	IS	High IS	4.897	3.925	2.157
	SA	High SA	4.125	3.153	1.895
Outcome variables	CDE	High CDE	4.579	3.475	1.933
		Non-high CDE	1.933	3.475	4.579

CDE, corporate digital entrepreneurship; OI, organizational inertia; DC, digital capability; EC, entrepreneurial culture; IS, institutional support; SA, strategic alliance.

Then we analyzed the necessary conditions for CDE for high/non-high-level before conducting configuration analysis. The results showed (Table 7) that the consistency of non-high OI (~OI) exceeded the 0.9 level, indicating that non-high OI was a necessary condition for the formation of high-level CDE; No conditional variable was necessary for the formation of a non-high-level CDE.

**Table 7:** The analysis of the necessary conditions for CDE.

Condition variables		Outcome variables	
		High CDE	Non-high CDE
OI	OI	0.246	0.873
	~OI	**0.914**	0.361
DC	DC	0.617	0.172
	~DC	0.294	0.625
EC	EC	0.725	0.106
	~EC	0.268	0.673
IS	IS	0.628	0.149
	~IS	0.198	0.564
SA	SA	0.685	0.484
	~SA	0.274	0.662

~ represents the “NO” of logical operations; CDE, corporate digital entrepreneurship; OI, organizational inertia; DC, digital capability; EC, entrepreneurial culture; IS, institutional support; SA, strategic alliance.

Finally, based on the necessary condition analysis, we incorporated other conditional variables into the analytical framework to explore the conditional configuration for the formation of high-level CDE. We used the fsQCA3.0 software to set the consistency threshold of the solution to 0.8, and selected the frequency bit to be 1. The results suggested (see Table 8) that there were four configurations that generated high-level CDE, namely H1: ~OI*DC*EC*SA, H2: ~OI*DC*SA, H3: ~OI*EC*SA, H4: ~OI*DC*EC. The consistency indicators of the above configurations were 0.937, 0.926, 0.912, and 0.909, respectively, indicating high consistency. The consistency and coverage of the model solutions were 0.916 and 0.829, indicating that they were sufficient conditions to promote CDE, and explained the main reasons for high-level CDE strongly. In addition, it also indicated that OI had a hysteresis effect on CDE, and the four factors of organization and environment were not necessary conditions to lead to high-level CDE.

**Table 8:** The configuration conditions for the formation of non-high CDE.

Condition variables	High CDE			
	*H1*	*H2*	*H3*	*H4*
OI	⊗	⊗	⊗	⊗
DC	●	●		●
EC	●		●	●
IS				
SA	●	●	●	
Consistency	0.937	0.926	0.912	0.909
Coverage	0.601	0.593	0.587	0.564
Unique coverage	0.112	0.095	0.093	0.092
Solution consistency	0.916			
Solution coverage	0.829			

● Indicates that the core condition appears; ⊗ Indicates that the core condition does not appear; ● Indicates that the edge condition appears; ⊗ Indicates that the edge condition does not appear; a space indicates that the variable is optional; CDE, corporate digital entrepreneurship; OI, organizational inertia; DC, digital capability; EC, entrepreneurial culture; IS, institutional support; SA, strategic alliance.

## 6. Discussion

### 6.1. Discussion of results

First, this study confirms that OI is negatively associated with CDE. On the one hand, the results of fsQCA reveal that non-high level OI (~OI) is a necessary condition for the formation of high-level CDE. In other words, the absence of OI is the core condition for high-level CDE. On the other hand, the results of multiple regression analysis also discover that the impact of OI on CDE is significantly negative. This result is similar to that of Wang et al. (2021) and shows that firms’ OI is negatively correlated to entrepreneurial orientation. These findings contribute substantially to the identification of antecedent variables that inhibit the healthy development of CDE and increase the success rate of CDE. Specifically, with the growth and development of firms, OI would prevent organizations and members from taking immediate perception and response to the changes in the external competitive environment and inhibit CDE.

Second, this study demonstrates that DC, EC, and SA could mitigate the inhibitory effect of OI on CDE. On the one hand, the results from multiple regression analysis prove that both DC, EC, and SA negatively moderated the relationship between OI and CDE. However, the regression coefficient of the interaction between IS and OI is not significant. On the other hand, the results from fsQCA also demonstrate that IS is an optional variable in the four configurations that produce high levels of CDE. This conclusion indicates that IS lacks direct effects in promoting CDE and overcoming the inhibition effect of OI. Moreover, it supports the findings of previous studies (Lukman et al., 2021), which suggests that institutional support plays a moderation role between attitude and social entrepreneurship intention. One possible reason is that IS is a favorable factor in the macro or industrial environment. Whether through the resource path or the information path, the alleviating or eliminating of the effect of IS on OI is not directly generated, and there may also be mediation links such as absorptive capability, organizational learning, and other organizational behaviors or activities. Therefore, the moderating effect of IS is not supported in the relationship between OI and CDE.

Third, this study reveals that the moderating effects of DC, EC, and SA show different characteristics after dividing OI into insight inertia, action inertia, and psychological inertia. DC only moderates the relationship between action inertia and CDE, which implies that outstanding DC could help organizations and employees to take immediate response to digital transformation, application, and innovation. This is in line with the study results of Rupeika-Apoga et al. (2022) showing that digital capability has a positive direct effect on digital transformation. Moreover, Saputra et al. (2022) found that digital capability plays a strategic role in supporting top management in applying ambidextrous leadership in leading organizations during turbulent times. However, the moderating effect of DC is not significant on the dimensions of insight inertia and psychological inertia. EC moderates the relationship between action inertia and CDE and the relationship between psychological inertia and CDE, which suggests that EC encouraging creativity and risk-taking could not only inspire incumbent firms to seize digital opportunities but also alleviate employees’ psychological anxiety and resistance when they are confronted with uncertainties about the application of digital technologies. Kayed et al. (2022) also shows the culture has a significant influence on entrepreneurial intention through psychological empowerment. SA moderates the relationship between insight inertia and CDE and the relationship between action inertia and CDE, which indicates that SA based on shareholding or joint investment could help leading incumbent firms absorb external knowledge and information and introduce external perspectives to stimulate employees’ interest and creativity in digital entrepreneurship.

### 6.2. Theoretical contributions

This research has threefold theoretical contributions. First, this study enriches and extends the literature on corporate entrepreneurship in the digital era by conceptualizing CDE as the fusion of corporate entrepreneurship and digital entrepreneurship. Specifically, previous studies have failed to identify the unique feature of CDE, which results in confusing the conception of CDE with traditional corporate entrepreneurship and digital entrepreneurship (Li, 2021; Li et al., 2022). By defining the dimensions and developing a scale of CDE, this study expands the research scope of digital entrepreneurship and improves the conceptual framework of corporate entrepreneurship in the prosperous digital era.

Second, this research plugs the gap left behind by previous studies that have not advanced the knowledge on identifying the antecedent variables which could be negatively connected with CDE, such as OI. Unlike those studies focusing on the antecedent variables that have positive impacts on CDE, this study proves the negative impact of OI on CDE from the perspective of reducing the failure rate. This finding not only responds to the argument that OI could be a major hindrance to organizational change and innovation (Godkin and Allcorn, 2008; Moradi et al., 2021), but also enriches the literature on the antecedent factors in influencing CDE.

Third, this research also fills the gap that previous studies have largely ignored the conditions under which OI hinders CDE, thus failing to understand when enterprises could eliminate the inhibition effect of OI on CDE. This study clarifies the different eliminating mechanisms in the relationship between OI and CDE by discussing the moderating effects of DC, EC, IS, and SA. These findings answer the question of “How to fuel CDE by combing different types of conditions with OI.”

### 6.3. Managerial implications

Our findings offer some valuable managerial implications. On the one hand, incumbent firms need to take a hard look at the scope and extent to which existing experience generates value. When organizational innovation and creation are needed, enterprises need to recognize the harm of OI and strive to overcome it through orderly management actions. On the other hand, OI is composed of different contents, and different enterprises may have different types of inertia, so it is necessary to take targeted management measures to overcome OI effectively. Specifically, enterprises facing insight inertia need to cultivate EC and build SA with investment as the core to help them promptly identify and accurately recognize the external environment changes. In addition to building an EC and SA, enterprises should also cultivate DC at the organizational and employee levels when action inertia exists with enterprises. When enterprises need to overcome psychological inertia, cultivating EC is a crucial method to reduce the psychological anxiety and defense of organization members and actively participate in digital innovation and value creation. Finally, the fsQCA is able to explore the configuration effects between variables, which helps managers to effectively configure various resources to realize CDE if they are not clear about the causal relationship between variables.

### 6.4. Limitations and future research

This study presents some limitations which may act as starting points for future empirical studies. First, this research only focuses on discussing the moderating effects of DC, EC, IS, and SA in the relationship between OI and CDE, but there are still some core moderators, such as absorptive capability and technological spillover. Thus, future research needs to explore these moderating effects. Second, this study ignores exploring the mechanism of the relationship between OI and CDE. Some mediators, such as resource integration, innovation strategy, and knowledge management, need to be further discussed. Third, although empirical studies can help understand the causal relationship between OI and CDE, this approach is usually based on static analysis. Future research will need to adopt case studies to identify differences in the different stages and dimensions of CDE and how to deal with OI.

## Data availability statement

The original contributions presented in the study are included in the article/Supplementary material, further inquiries can be directed to the corresponding author/s.

## Author contributions

WL: writing the manuscript and mentoring. WC: planning the study and writing the manuscript. QP: reviewing the manuscript and providing language services. JS: data analysis and providing the methodology. All authors contributed to the article and approved the submitted version.

## Funding

The study was funded by the National Natural Science Foundation of China (NO. 71872024), Science and Technology Research Program of Chongqing Municipal Education Commission (NO. KJQN202101101), Humanity and Social Science Research Project of Chongqing Municipal Education Commission (NO. 21SKGH171), and Graduate Research and Innovation Foundation of Chongqing Education Commission (NO. CYS22647).

## Conflict of interest

The authors declare that the research was conducted in the absence of any commercial or financial relationships that could be construed as a potential conflict of interest.

## Publisher’s note

All claims expressed in this article are solely those of the authors and do not necessarily represent those of their affiliated organizations, or those of the publisher, the editors and the reviewers. Any product that may be evaluated in this article, or claim that may be made by its manufacturer, is not guaranteed or endorsed by the publisher.

## Footnotes

^1^A listed company located in Shenzhen and committed to becoming an international leading provider of personal financial services.

^2^A listed company located in Chongqing and seeking to build intelligent communities and cities by using digital technologies.
