# Supplementary material for: How to mitigate the inhibitory effect of organizational inertia on corporate digital entrepreneurship?
Source: Front Psychol. 2023 Mar 9;14:1130801. doi: 10.3389/fpsyg.2023.1130801 (PMC10035578; doi:10.3389/fpsyg.2023.1130801)
Supplement: Supplementary file 1 [file Data_Sheet_1.pdf]

Appendix I: Questionnaire

**Organizational inertia** (Huang et al., 2013)					
Insight inertia					
- Our company has difficulty identifying how other firms solve problems. (II01)	1	2	3	4	5
- Our company rarely observes changes in the external environment. (II02)	1	2	3	4	5
- Our company mainly uses existing information and knowledge to solve problems. (II03)	1	2	3	4	5
- Our company rarely try to observe and learn new ideas and methods to change existing patterns or process. (II04)	1	2	3	4	5
Action inertia					
- Our company has a deep-rooted organizational culture. (AI01)	1	2	3	4	5
- Our company values are sacred and we are absolutely not going to change them. (AI02)	1	2	3	4	5
- Our company will follow the suggestions and requirements of others to change my methods for solving problems. (AI03)	1	2	3	4	5
- Past knowledge and experience can increase the employees' work efficiency. (AI04)	1	2	3	4	5
- When our companies ask employees to change their behavior, they are often met with great resistance. (AI05)	1	2	3	4	5
Psychological inertia					
- Employees feel threatened by any organizational changes. (PI01)	1	2	3	4	5
- Employees feel defensive when there are any organizational changes. (PI02)	1	2	3	4	5
- Employees feel anxious when they recall painful past experiences arising from change. (PI03)	1	2	3	4	5
- Company employees like the current processes and do not like change. (PI04)	1	2	3	4	5
**Corporate digital entrepreneurship** (Li et al., 2022)					
Digital strategy generation					
- Our company uses the digital technology to identify new target markets. (DSG01)	1	2	3	4	5
- Our company uses the digital technology to change the way we compete. (DSG02)	1	2	3	4	5
- Our company integrates digitalization into its organizational vision or strategic goals. (DSG03)	1	2	3	4	5
- Our company has restructured its original business for the use of the digital technology. (DSG04)	1	2	3	4	5
Digital innovation					
- In order to use the digital technology, our company has changed its organizational structure, system and culture. (DI01)	1	2	3	4	5
- Our company uses the digital technology to innovate existing products/services. (DI02)	1	2	3	4	5
- Our company uses the digital technology to improve work or productivity. (DI03)	1	2	3	4	5
- Our company has used the digital technology to revolutionize its internal business processes. (DI04)	1	2	3	4	5
Digital business development					
- Our company has established a new business division related to the application of the digital technology. (DBD01)	1	2	3	4	5
- Our company acquires the digital technology or business through mergers and acquisitions. (DBD02)	1	2	3	4	5
- Our company is constantly investing in the development of the digital technology. (DBD03)	1	2	3	4	5
- Our company is actively applying the digital technology to new business development. (DBD04)	1	2	3	4	5
**Digital capability** (Proksch et al., 2021)					
- Our company adapts digital offerings whenever changing business needs arise. (DC01)	1	2	3	4	5
- Our company implements new digital products and services on a regular basis. (DC02)	1	2	3	4	5
- Our IT integrates the most current digital offerings by third parties like digital payments, customer relationship management systems, and others. (DC03)	1	2	3	4	5
- Our company provides access to a variety of digital devices. (DC04)	1	2	3	4	5
- Our company stores and shares all operational and administrative data digitally. (DC05)	1	2	3	4	5
- Our company uses the most current IT infrastructure. (DC06)	1	2	3	4	5

- Our company have Internet access with gigabit speed. (DC07)	1	2	3	4	5
- Digital skills are an important selection criterion in recruiting new team members. (DC08)	1	2	3	4	5
- Our company offers different trainings (courses, literature, coaching) to improve the digital expertise of our team members. (DC09)	1	2	3	4	5
- Our team members use all digital services and products our company offer. (DC10)	1	2	3	4	5
- Our team has the necessary skills to further digitalize our company. (DC11)	1	2	3	4	5
- Our company actively discusses our digital projects within our company including failures and best practices. (DC12)	1	2	3	4	5
**Entrepreneurial culture** (Buccieri et al., 2020)					
- The company's management team likes risky projects with the chance of a high return. (EC01)	1	2	3	4	5
- The company's management team decides that taking bold and broad action is the best way to achieve its marketing goals. (EC02)	1	2	3	4	5
- Our company is often the first in the industry to launch a new product/service of its kind. (EC03)	1	2	3	4	5
- Our company has continued access to resources for new business without annual budget constraints. (EC04)	1	2	3	4	5
**Institutional support** (Smirnova, 2020)					
During the last three years, the government, industry groups and other relevant agencies:					
- Implemented policies and programs that have been beneficial to the application and innovation of digital technologies in enterprises. (IS01)	1	2	3	4	5
- Provided needed technology information and technical support for digital application. (IS02)	1	2	3	4	5
- Played a significant role in providing financial support for digital applications in enterprises. (IS03)	1	2	3	4	5
- Helped our company to obtain digital technology, intelligent manufacturing and other related technical resources. (IS04)	1	2	3	4	5
**Strategic alliance** (Cacciolatti et al., 2020)					
- Our company acquires digital technology resources through investment or shareholding. (SA01)	1	2	3	4	5
- Our company develops digital products or services through joint ventures. (SA02)	1	2	3	4	5
- Our company enters new industries or markets by forming joint ventures. (SA03)	1	2	3	4	5
- Our company acquires new customers through investment or shareholding. (SA04)	1	2	3	4	5
